# Construction and application of a predictive model for optimal peripherally inserted central catheter (PICC) insertion depth in preterm infants under high-frequency ultrasound guidance based on clinical parameters

**DOI:** 10.1371/journal.pone.0332447

**Published:** 2025-09-16

**Authors:** Wenlan Li, Bo Gou, Xin We, Jicheng Zhang, Bentian Liu, Zhuyu Zhou, Jian Liu

**Affiliations:** 1 Department of Ultrasound, The First Affiliated Hospital of Chengdu Medical College, Chengdu, China; 2 Department of Ultrasound, Deyang People’s Hospital, Deyang, China; Baylor College of Medicine, UNITED STATES OF AMERICA

## Abstract

**Objective:**

This study aims to construct and validate a predictive formula based on routine clinical parameters for determining the optimal catheter placement depth (OCPD) in preterm infants undergoing peripherally inserted central catheter (PICC) insertion via the basilic vein (BV) or axillary vein (AXV). The goal is to provide a standardized reference protocol for precise PICC placement in neonatal intensive care, with the aim of enhancing procedural accuracy and reducing catheter-related complications.

**Methods:**

This prospective study enrolled 105 preterm infants, who were categorized into two groups based on the puncture site: the basilic vein PICC group (BV-PICC, n = 59) and the axillary vein PICC group (AXV-PICC, n = 46). All catheter placements were performed under high-frequency ultrasound guidance to ensure accurate positioning and optimal catheter depth. The optimal catheter insertion length was recorded for each infant. Subsequently, clinical data were collected and analyzed, including gestational age, head circumference, chest circumference, maximum abdominal circumference, mid-forearm circumference, body weight, and body length. Multiple linear regression analysis was conducted to explore the relationship between optimal catheter depth and the clinical parameters of the preterm infants. Based on this analysis, a predictive formula for PICC insertion depth in preterm infants was developed using clinical parameters.

**Results:**

In the BV-PICC group, birth weight and weight at the time of catheterization were identified as significant predictors. The optimal catheter placement depth (cm) was calculated using the following formula: OCPD = 8.205 + 0.005 × birth weight (g)–0.003 × weight at catheterization (g). In the AXV-PICC group, only body length at catheterization was identified as a significant predictor. The formula was: OCPD = 1.024 + 0.177 × body length at catheterization (cm).

**Conclusion:**

The predictive formulas for the OCPD of PICC inserted via different pathways in preterm infants, developed under high-frequency ultrasound guidance and based on clinical parameters, demonstrated accuracy and excellent clinical applicability. These formulas provide a valuable reference for both standardized and individualized PICC placement in preterm infants, thereby facilitating evidence-based clinical decision-making and potentially reducing catheter-related complications.

## 1 Introduction

With continuous advancements in medical technology, the survival rate of preterm infants has significantly improved. However, due to their low gestational age and birth weight, these infants often experience multiple complications, such as respiratory distress syndrome, intestinal problems, retinopathy, infections, and jaundice [1–4], necessitating prolonged intravenous nutritional support and pharmacological treatment. In this context, the effective and safe management of venous access in preterm infants has become a crucial clinical issue. Peripherally inserted central catheters (PICC) have been widely used in preterm infants due to their relatively lower risk of infection and prolonged indwelling time [5,6]. However, traditional PICC insertion methods face dual challenges of accuracy and risk of complications, which are particularly pronounced in extremely low birth weight preterm infants [7].Currently, clinical methods for predicting and confirming PICC tip placement primarily include surface landmark methods, X-ray localization, ultrasound guidance, electrophysiological navigation, and venography, each with its own advantages and limitations [8–11]. Ultrasound, with its advantages of being radiation-free, real-time monitoring, and high accuracy, is particularly suitable for PICC placement in preterm infants [12]. High-frequency ultrasound probes provide high-resolution imaging, which is particularly advantageous for visualizing small neonatal blood vessels and nerve structures, thereby enhancing catheter placement accuracy and safety [13]. Ultrasound-guided PICC placement generally involves estimating the insertion depth using surface anatomical landmarks and formula-based predictions, followed by catheter insertion and real-time ultrasound-guided tip positioning and adjustment. The higher the accuracy of the insertion depth prediction formula, the greater the first-attempt success rate. However, existing formulas for predicting insertion depth vary in included parameters, usability, and simplicity, and their accuracy remains unverified, limiting their clinical applicability [14–18].

This study aims to collect clinical data from preterm infants undergoing PICC placement via the basilic vein (BV) or axillary vein (AXV) and determine tip positioning using high-frequency ultrasound. We will analyze the correlation between body parameters and the optimal catheter placement depth (OCPD) and establish a predictive formula for OCPD in preterm infants undergoing upper limb venous PICC placement. The goal is to improve placement accuracy and convenience, providing a reference for precise PICC insertion in preterm infants.

## 2 Materials and methods

### 2.1 Study subjects

Preterm infants who underwent PICC placement via BV or AXV in the neonatal intensive care units (NICUs) of The First Affiliated Hospital of Chengdu Medical College and Deyang People’s Hospital from December 2022 to May 2024 were included in this study. Inclusion criteria: (1) gestational age < 37 weeks at birth; (2) meeting the indications for PICC placement; (3) informed consent obtained from guardians after explaining the study’s purpose and procedures. Exclusion criteria: (1) unsuccessful catheter insertion via the aforementioned veins; (2) infants with fever, bacteremia, coagulation disorders, or limb movement disorders; (3) neonates with congenital malformations. The study was approved by the hospital ethics committee (Ethics Approval Number:2021-06-054-K01). The recruitment period for this study was from January 2022 to May 2024. Informed consent was obtained from the parents or legal guardians of all participating children prior to their inclusion in the study.

### 2.2 Study methods

A GE VIVID IQ color Doppler ultrasound system (General Electric, USA) was used, equipped with a 7−15 MHz high-frequency linear probe (Model S5-1). A 1.9F neonatal PICC catheter (BD, USA) was used for catheterization.

According to the puncture site, catheterization routes were classified as either via the basilic vein (BV-PICC) or the axillary vein (AXV-PICC). The detailed procedural steps are outlined as follows: (1) Positioning: The infant was placed in the supine position. (2) Ultrasound Examination: Routine cardiac structure assessment was performed, along with evaluation of the superior vena cava (SVC) for abnormalities. (3) Catheterization: The BV or AXV was selected as the target puncture site for ultrasound-guided PICC placement. For BV-PICC placement, the puncture site was located in the mid-upper arm, corresponding to zone II of the Dawson method [19]. For AXV-PICC placement, the catheter was inserted in the proximal segment of the axillary vein near the axilla, where vascular visualization was optimal under ultrasound. After identifying the target vessel under ultrasound, standardized percutaneous puncture techniques were employed. Upon successful puncture, the catheter was gently advanced along the natural vascular pathway. The catheter exit site was consistently defined as the point of needle entry through the skin, to ensure standardization and consistency in measuring catheter depth across all cases. (4) Ultrasound Localization: A high-frequency probe was placed in the sagittal plane at the subxiphoid midline position, using the liver as an acoustic window for subcostal scanning, or in the sagittal plane at the parasternal line under the clavicle for scanning. The PICC tip was identified as a high-echo “equal sign” structure within the SVC, with the ideal tip position located 0-2.0 cm from the entrance of the right atrium [20–22]. The catheter insertion length corresponding to the optimal tip position was recorded for each preterm infant. (5) Collect and record the clinical data of all preterm infants, including gestational age, head circumference, chest circumference, maximal abdominal circumference, mid-forearm circumference, body weight, and body length.

All ultrasound examinations and PICC placements were performed by the same associate chief physician from the ultrasound department, and data were measured three times and averaged.

### 2.3 Observational indicators

Basic information of the preterm infants and the catheterization depth under high-frequency ultrasound were recorded. gestational weeks; head circumference, chest circumference, maximum abdominal circumference, and forearm circumference on the day of catheterization; the weight of the preterm infants on the day of catheterization (i.e., weight at catheterization), and the body length of the preterm infants on the day of catheterization (i.e., body length at catheterization).

### 2.4 Statistical analysis

SPSS 27.0 software was used for data analysis. Quantitative data following normal or approximately normal distribution were expressed as mean ± standard deviation (s). Pearson correlation analysis was used for normally distributed data to assess the relationship between ultrasound-determined insertion depth and clinical parameters, while Spearman correlation analysis was applied for non-normally distributed data. Multiple linear regression analysis was conducted to explore the relationship between insertion depth and general parameters, and a predictive model for catheter insertion depth was constructed. The significance of correlation and regression coefficients was tested using t-tests, while the significance of the regression equation was assessed using F-tests. A *P*-value <0.05 was considered statistically significant.

## 3 Results

### 3.1 General data

A total of 105 preterm infants were included in this study. Based on different puncture sites, they were divided into the BV-PICC group (n = 59) and the AXV-PICC group (n = 46) (Table 1).

**Table 1:** General characteristics of preterm infants undergoing upper limb vein PICC placement.

Variable	BV-PICC Group (n = 59)	AXV-PICC Group (n = 46)	Mean ± Standard Deviation (n = 105)
Gestational age (weeks)	31.56 ± 2.12	31.57 ± 2.04	31.51 ± 2.10
Body length at catheterization (cm)	40.5 ± 3.1	41.3 ± 3.8	40.8 ± 3.4
Birth weight (g)	1505.29 ± 306.29	1613.17 ± 482.66	1539.64 ± 388.00
Weight at catheterization (g)	1423.12 ± 297.71	1519.23 ± 517.37	1451.85 ± 400.94
Head circumference (cm)	28.5 ± 2.2	28.4 ± 2.0	28.5 ± 2.3
Chest circumference (cm)	25.9 ± 2.5	26.1 ± 2.9	25.9 ± 2.6
Maximal abdominal circumference (cm)	24.8 ± 3.1	24.6 ± 2.9	24.5 ± 3.0
Upper limb forearm circumference (cm)	7.5 ± 1.1	7.2 ± 1.2	7.3 ± 1.1

### 3.2 Correlation between optimal catheter placement depth and physical parameters

In the BV-PICC group, birth weight, gestational age, weight at catheterization, and forearm circumference followed a normal distribution, whereas body length at catheterization, head circumference, chest circumference, maximal abdominal circumference, and umbilical-abdominal circumference did not. Pearson and Spearman correlation analyses showed that the optimal catheter placement depth determined by high-frequency ultrasound was significantly positively correlated with birth weight (r = 0.351), gestational age (r = 0.283), weight at catheterization (r = 0.264), body length at catheterization (*r = 0.378), head circumference (*r = 0.244), chest circumference (*r = 0.385), maximal abdominal circumference (*r = 0.256), and umbilical-abdominal circumference (*r = 0.203) (*P* < 0.05).

In the AXV-PICC group, gestational age and body length at catheterization followed a normal distribution, while birth weight, weight at catheterization, forearm circumference, head circumference, chest circumference, maximal abdominal circumference, and umbilical-abdominal circumference did not. Pearson and Spearman correlation analyses showed that the optimal catheter placement depth determined by high-frequency ultrasound was significantly positively correlated with body length at catheterization (r = 0.283), gestational age (r = 0.281), birth weight (*r = 0.544), head circumference (*r = 0.434), chest circumference (*r = 0.507), maximal abdominal circumference (*r = 0.472), umbilical-abdominal circumference (*r = 0.487), weight at catheterization (*r = 0.516), and forearm circumference (*r = 0.340) (*P* < 0.05) (Table 2).

**Table 2:** Correlation analysis of catheterization depth through upper limb vein under high-frequency ultrasound and general characteristics.

Variable	BV-PICC		AXV-PICC	
	r	*P*	r	*P*
Birth weight	0.351	0.000^**^	0.544^*^	0.000^**^
Gestational age	0.283	0.000^**^	0.281	0.006^**^
Weight at catheterization	0.264	0.000^**^	0.516^*^	0.000^**^
Forearm circumference	−0.163	0.078	0.340^*^	0.003^**^
Body length at catheterization	0.378^*^	0.000^**^	0.283	0.006^**^
Head circumference	0.244^*^	0.000^**^	0.434^*^	0.000^**^
Chest circumference	0.385^*^	0.000^**^	0.507^*^	0.000^**^
Maximal abdominal circumference	0.256^*^	0.000^**^	0.472^*^	0.000^**^
Umbilical-abdominal circumference	0.203^*^	0.028^*^	0.487^*^	0.001^**^

*P < 0.05, **P < 0.001, r: Pearson correlation coefficient, *r: Spearman correlation coefficient.

### 3.3 Prediction formula for optimal PICC placement depth using high-frequency ultrasound

**Fig 1:**
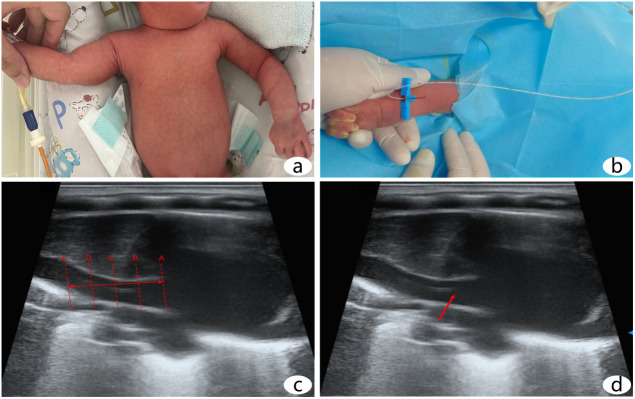
High-frequency ultrasound-guided upper limb PICC placement. a: The infant in the supine position with the right basilic vein selected; b: PICC placement guided by high-frequency ultrasound via the basilic vein (arrow indicates catheter); c: Ultrasound image of optimal catheter placement depth (A-line: superior vena cava entrance to the right atrium; B-line: 0.5 cm above the entrance; C-line: 1 cm above the entrance; E-line: 2 cm above the entrance; A-E represent the acceptable range, with B-line being ideal; < E-line is too shallow, > A-line is too deep); d: Ultrasound image of the catheter tip (arrow indicates the tip).

In the BV-PICC group, birth weight and weight at catheterization were identified as significant variables through stepwise linear regression analysis, where the optimal catheter placement depth determined by high-frequency ultrasound was the dependent variable. The regression equation was established as: Optimal catheter placement depth (cm) = 8.205 + 0.005 × birth weight (g) – 0.003 × weight at catheterization (g). (Fig 1 and Table 3)

**Table 3:** Stepwise linear regression analysis of the catheterization depth in the basilic vein of the upper limb under high – frequency ultrasound positioning and body parameters.

Variable	Coefficient	Standard Error	Standardized Coefficient	t-value	*P*-value	95% CI	
						Lower	Upper
Constant	8.205	0.133	–	11.065	0.000	6.736	9.674
Birth weight	0.005	0.001	0.824	3.404	0.001	0.002	0.008
Weight at catheterization	−0.003	0.001	−0.507	−2.092	0.039	−0.006	0.000

In axillary vein peripherally inserted central catheter (AXV-PICC) placement, we performed stepwise linear regression analysis using birth weight, gestational age, catheterization weight, catheterization length, head circumference, chest circumference, maximum abdominal circumference, umbilical-level abdominal circumference, and forearm circumference as independent variables, with high-frequency ultrasound-localized insertion depth as the dependent variable. The final regression model retained only catheterization length as a significant predictor. The predictive equation for ultrasound-localized insertion depth was: Insertion depth (cm) = 1.024 + 0.177 × body length at catheterization (cm). (Table 4)

**Table 4:** Stepwise linear regression analysis of high-frequency ultrasound-localized catheter insertion depth versus anthropometric parameters in upper limb axillary venous access.

Independent Variable	Partial Regression Coefficient	Standard Error	Standardized β	t-value	*P*-value	95% CI	
						Lower	Upper
Constant	1.024	1.550	–	1.828	0.008	−1.801	3.857
Body length at catheterization	0.177	0.037	0.331	2.818	0.006	0.071	0.281

R = 0.283, R^2^ = 0.181, Adjusted R^2^ = 0.166, F = 18.258, Durbin-Watson = 1.889, *P* = 0.008.

## 4 Discussion

In neonatal, particularly preterm infant peripherally inserted central catheter (PICC) placement, the accuracy of catheter positioning is crucial. Improper catheter placement may lead to complications such as thrombosis, catheter migration, and puncture-related infections, which not only affect treatment efficacy but also pose serious risks to neonatal health [5,23]. Therefore, ensuring the optimal catheter tip position and determining the correct insertion depth are essential for reducing catheter-related complications [24].Currently, several methods are employed to determine PICC insertion depth, including anatomical landmark techniques, radiographic imaging (X-ray), electrophysiological navigation, venography, and ultrasound guidance [25]. The anatomical landmark and three-point positioning methods are simple to perform and do not require specialized equipment, making them particularly suitable for resource-limited settings [26]. However, their accuracy highly depends on the practitioner’s ability to correctly identify anatomical landmarks and may be affected by individual variability. Although radiographic imaging is widely recommended by guidelines as the gold standard for verifying catheter placement, it poses radiation risks, which may have potential health implications for vulnerable populations [27]. Intracavitary electrocardiographic (IC-ECG) localization has not been widely adopted in clinical practice, as it relies solely on P-wave variations to infer the proximity of the catheter tip to the right atrium, without providing direct anatomical visualization of the catheter tip location [28]. In contrast, ultrasound guidance, with its advantages of radiation-free imaging, applicability across all age groups, and real-time visualization of blood vessels and catheter positioning, has gained increasing clinical attention [29].

In this study, high-frequency ultrasound was used to determine PICC placement depth in 105 preterm infants via the Basilic Vein (BV) and axillary vein (AXV). The results indicated that optimal catheter placement depth (OCPD) was correlated with multiple physical parameters of preterm infants, consistent with previous studies [30 ,31]. Stepwise linear regression analysis revealed that for BV-PICC placement, the ultrasound-guided insertion depth could be estimated using the following formula: Insertion depth (cm) = 8.205 + 0.005 × birth weight (g) – 0.003 × weight at catheterization (g). Birth weight and weight at catheterization were identified as key variables determining insertion depth. Unlike traditional BV-PICC placement studies that primarily rely on body length as an anatomical reference [32], this study utilized high-frequency ultrasound and predictive modeling based on birth weight and catheterization weight. Since ultrasound can directly visualize the vascular structure and catheter movement, it minimizes uncertainties caused by individual anatomical differences [33,34]. Moreover, birth weight and catheterization weight better reflect neonatal physiological status and developmental level [35], as these parameters directly influence venous size and position [36,37]. This approach enhances the precision of insertion depth predictions and provides individualized reference values for catheterization in neonates with different birth weights, particularly for extremely low birth weight or low birth weight preterm infants [38,39]. Additionally, ultrasound technology, with its increasing clinical application, enables real-time visualization of catheter placement, allowing clinicians to adjust insertion depth accordingly and meet practical clinical demands.

For AXV-PICC placement, the regression equation derived in this study was: Insertion depth (cm) = 1.024 + 0.177 × body length at catheterization (cm). Previous research by Shao Zhenzhen [40] also demonstrated a positive correlation between neonatal body length and PICC insertion depth, with a regression model of: PICC length (cm) = 0.181 × body length (cm) + 1.015. The consistency between these findings enhances the reliability of our study and supports the use of body length as an effective parameter for adjusting PICC insertion depth. Body length is a simple and easily obtainable physiological metric that serves as a practical clinical reference, enabling physicians to more accurately predict and adjust catheterization depth [14,41,42]. This can effectively reduce the risks of catheter migration, thrombosis, and other complications. However, body length is not the sole influencing factor. In clinical practice, variations in neonatal body morphology, vascular conditions, and fluid balance [43–45] may also significantly impact PICC placement success and safety. For instance, preterm infants have thinner vascular walls [46] and lower vascular elasticity [47,48], which may lead to discrepancies between predicted and actual catheter insertion depth. Future studies should further explore the combined effects of multiple factors on PICC insertion depth and develop more precise and individualized predictive models.

This study has several limitations. First, the sample size was relatively small and limited to two medical centers, which may affect the generalizability of the findings. Second, the study primarily focused on static anthropometric parameters such as birth weight and body length, without evaluating dynamic physiological variables such as blood volume fluctuations or venous elasticity that may also influence optimal catheter depth. Lastly, long-term follow-up data regarding catheter-related complications and functional outcomes were not included. Future studies should involve larger, multicenter cohorts and incorporate multidimensional clinical and longitudinal data to further refine and validate the applicability of the predictive models in neonatal populations.

In summary, the predictive formulas for optimal PICC insertion depth in preterm infants based on clinical parameters under high-frequency ultrasound guidance provide a practical framework for both standardized and individualized catheter placement. These models have the potential to reduce complication rates and improve procedural success, and are thus worthy of broader clinical adoption.
